# Gross Domestic Product and Health Expenditure Growth in Balkan and East European Countries—Three-Decade Horizon

**DOI:** 10.3389/fpubh.2020.00492

**Published:** 2020-09-15

**Authors:** Milos Stepovic, Nemanja Rancic, Berislav Vekic, Viktorija Dragojevic-Simic, Stefan Vekic, Nenad Ratkovic, Mihajlo Jakovljevic

**Affiliations:** ^1^Faculty of Medical Science, University of Kragujevac, Kragujevac, Serbia; ^2^Centre for Clinical Pharmacology, Military Medical Academy, Belgrade, Serbia; ^3^Medical Faculty of the Military Medical Academy, University of Defence, Belgrade, Serbia; ^4^Clinic for Surgery, University Hospital Center “Dr. Dragisa Misovic”, Belgrade, Serbia; ^5^Department of Surgery, Faculty of Medical Sciences, University of Kragujevac, Kragujevac, Serbia; ^6^Faculty of Economics, University of Belgrade, Belgrade, Serbia; ^7^Treatment Sector, Military Medical Academy, Belgrade, Serbia; ^8^Institute of Comparative Economic Studies, Hosei University, Tokyo, Japan; ^9^The N.A.Semashko Public Health and Healthcare Department, I.M. Sechenov First Moscow State Medical University (Sechenov University), Moscow, Russia; ^10^Department of Global Health Economics and Policy, Faculty of Medical Sciences, University of Kragujevac, Kragujevac, Serbia

**Keywords:** health care, private health expenditure, public health expenditure, Balkan, Southeastern countries

## Abstract

**Background:** Dynamics of health care has changed over time along with development of the countries themselves. The aim of the study is to compare macroeconomic and health expenditure indicators of interest, such as total health expenditure (THE) as percentage of global domestic product, global domestic product per capita in US$, and private households' out-of-pocket payments of Balkan and Eastern European countries on health, as well as to assess their progress over the observed period.

**Methods:** This research report represents a descriptive data analysis of indicators extracted from the European Health for All database. The data were analyzed using a linear trend and regression analysis to estimate the timeline changes.

**Results:** Greece and Slovenia have the largest median values of global domestic product per capita throughout the whole period, and the largest increment trend was in Lithuania. Median value in out-of-pocket payment of THE was the highest in Albania and Ukraine, while the largest decrease in trend was noticed in Albania and Bosnia and Herzegovina. Bosnia and Herzegovina and Greece had the largest median value of THE as percentage of Gross Domestic Product (GDP) in the observed period, while regression trend analysis showed that Serbia had the largest increase. Most of the countries showed a significant correlation between observed indicators.

**Conclusion:** Trends in the economy must be constantly monitored due to the fact that the population is aging and non-communicable diseases are multiplying, which requires innovations in medical treatment and pharmaceutical development.

## Background

The health system must be adequately funded or “the holes will lead to a rapid sinking” (1). There are a lot of suggested options in order to increase funding, such as increment in Gross Domestic Product (GDP) investments, better organization of resource allocation in health, introducing taxes on harmful products, insurance options, etc. (1). Better communication between health policymakers and controllers of public spending would result in more sustainable system that covers the most or all of citizen health care needs. Many changes affect countries, whether they are richer or poorer. Poor countries face challenges to organize affordable and sufficient quality health services, while richer countries struggle with aging populations. Both need to keep health expenditures under control, while they are rising a lot (2, 3).

By the method of health system funding, European countries can be sorted into three groups. The first group would present the countries with the Beveridge model of funding based mostly on taxation. This model is found in Great Britain, Spain, and New Zealand (4). The second group with the Bismark model rely their health funds on social insurance. This model is represented in Germany, France, Belgium, the Netherlands, Japan, and Switzerland (5). The third group of countries with the Semashko model bases their health funds on health care provided by the government for its citizens (6). The last one is characteristic for Russia and most other post-communist countries as well as in the most of the Southeastern countries and Balkan countries. Since they have a common past, they really look like each other in terms of forming good health systems, which means that they look up to the Russian Federation as a country that has set up such a system (7). This health system undertook some changes and transitioned into the social insurance or mixed social insurance/taxation-based system (8, 9). The former Yugoslavia had this kind of combined health system, while many Eastern European countries (the Russian Federation, Latvia, and Estonia) adopted this system during the 1990s. Changes always lead to certain problems, mainly through increased expenditures and increased labor costs. This has further led to one of the most regressive forms of payment for health services, and that is direct payment by the patient causing further conflicts: inequality between rich and poor (10, 11).

Therefore, adaptations of health systems began, which certainly could not be universal, because different countries had different levels of development. Universal health coverage became the ultimate goal for each country but, so far, none of them fully succeed. Some of them had better ideas, but adaptation varied, mostly dependent on the decisions and policies of the government. Economic analysis and comparison studies become the most relevant tools for accessing the health situation. The results of these studies proved to be very important in decision making concerning health investments, but a lot of aspects must be covered and many other factors need to be considered for outcomes to lead to universal coverage of health costs (12). Communicable, non-communicable diseases, aging population, new medical interventions and medications, gender inequalities, different level of country development, economic investments, and many other factors need to be considered (13).

Total health care spending increased during the 1960s and 1970s, slowed in the 1980s, and rose again in 1990s. This upward trend has continued, but the average percentage increase in gross domestic product (GDP) varied. The largest was in Romania and Estonia, from an average of 5 to 11% of GDP. As the GDP for health increased, the public health spending followed that rise accordingly. In the mid-1980s, this trend slowed down, and in the late 1990s and 2000s, it reached on average 6%. In the most EU countries, it varied between 12 and 15% in 2010 (14). Many researches show the significant correlation between higher spending on health in the countries that invest higher percentage of GDP per capita in comparison to the lower-income countries (15–18).

The well-known trend of health care systems in Central and Eastern European countries of using private budgets to cover medical expenses is in particular caused by health policy aiming to reduce the burden of the health problems of patients. Therefore, the form of full or partial payments for some medicines is used (19). In 2004, OOP payments accounted for 73–98% of total private health expenditures, and those payments mostly included dentists, laboratory tests, private treatments, etc. Expenditures of inpatient care and medicine and outpatient care are on the trend rise in Belarus, Estonia, and Hungary, and those difficulties will affect the people with lower socioeconomic status, resulting in delayed or unutilized health care needs (20).

Because of similar history and, also, the similar health system between these countries, we conducted a short data report to investigate the progress of selected economic indicators and to find a possible relationship between them. By doing this, we have obtained data concerning health financing of these countries that have similar development levels. By observing positive trends and analyzing their progress, it looks like that countries with less success in health system financing are more likely to adopt patterns of government investments, in comparison to more developed countries.

The aim of this study was to compare the total health expenditure (THE) as percentage of GDP, GDP per capita in US$, and private households' out-of-pocket payments on health as percentage of THE between Balkan and East European countries. The progress over the period between 1990 and 2014 was assessed.

## Methods

This research report article represents a descriptive data analysis of macroeconomic and health expenditure indicators, extracted from European Health for All database (HFA-DB) (21). This database provides indicators for 53 countries and 153 health indicators. Member States of the WHO European Region have been reporting essential health-related statistics to the Health for All (HFA) family of databases since the mid-1980s, making it one of the oldest sources of data of WHO. As it is based on reported data, the HFA family of databases is also particularly valuable. HFA databases bring together the indicators that are part of major monitoring frameworks (https://gateway.euro.who.int/en/indicators/hfa_565-6710-total-health-expenditure-as-of-gdp-who-estimates/).

Indicator of interest—THE as percentage of GDP, GDP per capita in US$, and private households' out-of-pocket payments on health as percentage of THE were extracted from the database. It included the following Balkan and Southeastern European countries: Albania, Bulgaria, Bosnia and Herzegovina, Belarus, Greece, Croatia, North Macedonia, Montenegro, Romania, the Russian Federation, Serbia, Slovenia, Turkey, Estonia, Lithuania, Latvia, and Ukraine. Observation period was from 1990 to 2014.

The data will be analyzed using a linear trend estimate. It is a statistical technique that helps interpret data where a series of process measurements are treated as a time series, a trend estimate used to make and justify statements of tendencies in the data, linking the measurements to the times in which they occurred. Linear trend estimation will show the occurrence of possible patterns in the behavior of the variables over the observed time period and will provide information on whether the trend developed is positive, negative, or even absent. Trend results will be presented in tabular form. Linear trend and regression analysis were used to access the timeline changes in observed indicators for each country and to calculate the progress of these countries over time. Median operation and interquartile range 25th–75th percentile were used for better comparison of each country. Financial parameters are expressed in US currency.

The data were also analyzed using the statistical program SPSS version 20. Pearson's coefficient of correlation was used to determine the relationship between each group of indicators of interest. Statistically significant results had a *p* value of <0.01.

The data are anonymous and do not belong to individual citizens. Therefore, there is no question of protecting the privacy of the data. According to the International Ethical Guidelines for Biomedical Research involving Humans and Good Clinical Practice Guidelines, a study like this does not require consideration by the Ethics Committee;

https://cioms.ch/wp-content/uploads/2017/01/WEB-CIOMS-EthicalGuidelines.pdf;
https://www.ema.europa.eu/en/documents/scientific-guideline/guideline-good-clinical-practice-e6r2-4-step-2b_en.pdf.

## Results

The largest median value of THE as percentage of GDP expenditure in the observed period had Bosnia and Herzegovina (8.75%) and Greece (8.66%) and the smallest had Turkey (5.35%) and Romania (5.25%) (Table 1). Regression trend analysis showed that the trend had the biggest increase in Serbia (*y* = 0.2481*x* + 5.8157; *R*^2^ = 0.9101) and Romania (*y* = 0.125*x* + 3.5459; *R*^2^ = 0.8148). The biggest decrement was in Albania (*y* = −0.0856*x* + 7.1254; *R*^2^ = 0.7184) and North Macedonia (*y* = −0.1663*x* + 9.6064; *R*^2^ = 0.7607).

**Table 1:** Total health expenditure (THE) as % of GDP, OOP as % of THE and GDP, US$ per capita for first and last year of observation, median for entire observed period, interquartile range, and linear trend regression analysis for all 17 countries.

**TOTAL HEALTH EXPENDITURE AS % OF GDP**					
**Countries**	**1995**	**2014**	**Median**	**IQR**	**Linear trend regression analysis**
Ukraine	7.01	7.10	6.74	0.71	*y* = 106.32*x* + 326.67; *R*^2^ = 0.5747
Albania	6.60	5.88	6.11	0.73	*y* = 191.37*x* – 396.13; *R*^2^ = 0.9098
Bosnia and Herzegovina	9.03	9.57	8.75	1.44	*y* = 233.86*x* – 743.15; *R*^2^ = 0.9367
North Macedonia	8.39	6.48	8.07	1.98	*y* = 158.56*x* + 934.78; *R*^2^ = 0.8187
Belarus	6.74	5.69	6.20	0.64	*y* = 248.15*x* – 32.949; *R*^2^ = 0.7273
Bulgaria	4.75	8.44	6.83	1.33	*y* = 310.49*x* – 392.72; *R*^2^ = 0.8583
Republic of Serbia	6.51	10.37	8.47	3.41	*y* = 255.71*x* – 277.32; *R*^2^ = 0.8347
Romania	3.22	5.57	5.25	1.28	*y* = 416.12*x* – 1053.3; *R*^2^ = 0.8546
Montenegro	7.42	6.42	7.41	1.39	*y* = 362.46*x* – 1810.6; *R*^2^ = 0.9022
Russian Federation	5.36	7.07	5.89	1.48	*y* = 467.5*x* – 307.95; *R*^2^ = 0.6612
Turkey	2.51	5.41	5.35	0.76	*y* = 421.57*x* + 644.13; *R*^2^ = 0.8718
Latvia	5.76	5.88	6.18	0.52	*y* = 688.64*x* – 2526.1; *R*^2^ = 0.9035
Lithuania	5.37	6.55	6.33	0.71	*y* = 714.22*x* – 2791.9; *R*^2^ = 0.9373
Croatia	6.74	7.80	7.20	1.17	*y* = 594.94*x* – 384.77; *R*^2^ = 0.8566
Estonia	6.32	6.38	5.84	1.31	*y* = 858.95*x* – 3041.6; *R*^2^ = 0.9315
Slovenia	7.46	9.23	8.48	1.22	*y* = 998.66*x* + 437.89; *R*^2^ = 0.8428
Greece	8.27	8.08	8.66	1.22	*y* = 577.01*x* + 9833.5; *R*^2^ = 0.5097
**OOP AS % OF TOTAL HEALTH EXPENDITURE**					
**Countries**	**1995**	**2014**	**Median**	**IQR**	**Linear trend regression analysis**
Ukraine	24.5	46.2	40.4	6.5	*y* = 0.4967*x* + 34.019; *R*^2^ = 0.2683
Albania	74	49.9	53.6	12.9	*y* = −1.4176*x* + 73.573; *R*^2^ = 0.8595
Bosnia and Herzegovina	63.9	27.9	38.3	17.2	*y* = −1.7146*x* + 57.662; *R*^2^ = 0.811
North Macedonia	40.4	36.7	38.0	6.3	*y* = −0.4161*x* + 42.309; *R*^2^ = 0.3822
Belarus	18.6	32	19.7	10.5	*y* = 0.8578*x* + 11.278; *R*^2^ = 0.6824
Bulgaria	26	44.2	39.7	8.3	*y* = 0.8826*x* + 29.076; *R*^2^ = 0.8112
Republic of Serbia	29.4	36.6	30.3	6.5	*y* = 0.5286*x* + 26.169; *R*^2^ = 0.5727
Romania	25.5	18.9	19.2	2.1	*y* = −0.2542*x* + 22.764; *R*^2^ = 0.2566
Montenegro	30	42.8	30.0	10.1	*y* = 0.7421*x* + 25.053; *R*^2^ = 0.6234
Russian Federation	16.9	45.8	31.1	14.2	*y* = 1.4826*x* + 17.106; *R*^2^ = 0.905
Turkey	29.7	17.8	20.8	11.0	*y* = −0.8211*x* + 30.319; *R*^2^ = 0.7986
Latvia	33.7	35.1	38.5	7.7	*y* = −0.4417*x* + 43.44; *R*^2^ = 0.3311
Lithuania	22.4	31.3	26.5	5.5	*y* = 0.3348*x* + 23.567; R^2^ = 0.4398
Croatia	13.5	11.2	13.5	2.1	*y* = −0.1283*x* + 15.15; *R*^2^ = 0.1329
Estonia	10.2	20.7	19.8	5.4	*y* = 0.4607*x* + 13.362; *R*^2^ = 0.4489
Slovenia	11.2	21.1	11.8	0.5	*y* = 0.0334*x* + 11.477; *R*^2^ = 0.1484
Greece	43.4	34.9	34.7	9.3	*y* = −0.6735*x* + 42.28; *R*^2^ = 0.6471
**GDP, US$ PER CAPITA**					
**Countries**	1990-2000^*^	**2017**	**Median**	**IQR**	**Linear trend regression analysis**
Ukraine	1570	2640	1570	2029	*y* = 106.32*x* + 326.67; *R*^2^ = 0.5747
Albania	617	4538	2110	3361	*y* = 191.37*x* – 396.13; *R*^2^ = 0.9098
Bosnia and Herzegovina	318	5148	3186	3487	*y* = 233.86*x* – 743.15; *R*^2^ = 0.9367
North Macedonia	2354	5415	2620	2848	*y* = 158.56*x* + 934.78; *R*^2^ = 0.8187
Belarus	2125	5728	2252	4429	*y* = 248.15*x* – 32.949; *R*^2^ = 0.7273
Bulgaria	2367	8228	3031	5627	*y* = 310.49*x* – 392.72; *R*^2^ = 0.8583
Republic of Serbia	2197	5900	4130	3380	*y* = 255.71*x* – 277.32; *R*^2^ = 0.8347
Romania	1681	10,818	3164	7219	*y* = 416.12*x* – 1053.3; *R*^2^ = 0.8546
Montenegro	1627	7783	6550	3987	*y* = 362.46*x* – 1810.6; *R*^2^ = 0.9022
Russian Federation	3485	10,743	3794	7679	*y* = 467.5*x* – 307.95; *R*^2^ = 0.6612
Turkey	2794	10,546	5380	7680	*y* = 421.57*x* + 644.13; *R*^2^ = 0.8718
Latvia	2329	15,594	9668	10,691	*y* = 688.64*x* – 2526.1; *R*^2^ = 0.9035
Lithuania	2169	16,681	9241	11,060	*y* = 714.22*x* – 2791.9; *R*^2^ = 0.9373
Croatia	4795	13,383	11,346	8278	*y* = 594.94*x* – 384.77; *R*^2^ = 0.8566
Estonia	3044	19,705	12,595	13,335	*y* = 858.95*x* – 3041.6; *R*^2^ = 0.9315
Slovenia	10,691	23,594	19,726	12,432	*y* = 998.66*x* + 437.89; *R*^2^ = 0.8428
Greece	9600	18,613	17,976	9831	*y* = 577.01*x* + 9833.5; *R*^2^ = 0.5097

*IQR, Interquartile range 25th−75th percentile*;

* *First year of following (1990–2000)*.

*Total health expenditure as % of GDP—THE as percent of GDP expenditure; OOP as % of total health expenditure—private households' out-of-pocket payments on health as percentage of THE; GDP, US$ per capita—gross domestic product, US$ per capita*.

The biggest median percentage in private households' out-of-pocket payments on health as percentage of THE (OOP payment of THE) came from Albania (53.6%) and Ukraine (40.4%) and the smallest percentages were noticed in Slovenia (11.8%) and Croatia (13.5%). Looking at the regression analysis, trend lines vary from country to country in the observed period. The biggest decrease was noticed in Albania (*y* = −1.4176*x* + 73.573; *R*^2^ = 0.8595) and Bosnia and Herzegovina (*y* = −1.7146*x* + 57.662; *R*^2^ = 0.811). The largest increase was noticed in Russia (*y* = 1.4826*x* + 17.106; *R*^2^ = 0.905) and Bulgaria (*y* = 0.8826*x* + 29.076; *R*^2^ = 0.8112) (Table 1).

**Graph 1:**
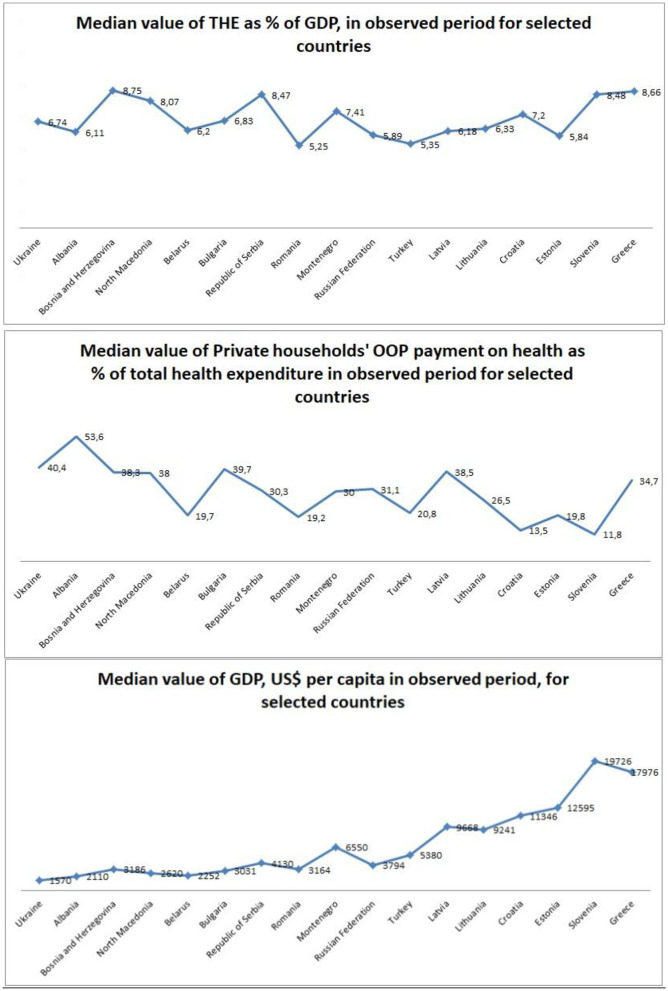
Median values for economic indicators in observed period for selected countries, shown as trend graphics.

Greece and Slovenia have the largest median values of GDP, US$ per capita throughout that period. This indicator for Greece was US$17,976 and that for Slovenia was US$19,726, while the smallest numbers were recorded in Ukraine (US$1570) and Albania (US$2110). Looking at the regression analysis, all countries showed increase over the observed time, with the biggest increase in Lithuania (*y* = 714.22*x* – 2791.9; *R*^2^ = 0.9373) and Bosnia and Herzegovina (*y* = 233.86*x* – 743.15; *R*^2^ = 0.9367) (Table 1 and Graph 1).

Pearson's correlation indicated that from 17 considered countries (Table 2), a total of 11 showed existence of significant positive (Albania, North Macedonia, Bulgaria, Republic of Serbia, and the Russian Federation) or negative (Romania, Montenegro, Turkey, Croatia, Estonia, and Greece) correlation between indicators THE as percentage of GDP expenditure and private households' out-of-pocket payments on health as percentage of THE. In Albania, North Macedonia, Bulgaria, Republic of Serbia, and the Russian Federation, growth of THE is in positive correlation with OOP, meaning that THE grows along with growth of OOP (correlation range from 0.51 in the Russian Federation to 0.87 in Albania). Opposite from this, negative correlation exists in Romania, Montenegro, Turkey, Croatia, Estonia, and Greece, meaning that with OOP growth, THE decreases (−0.42 in Estonia to −0.82 in Montenegro). The establishing relationship of THE as percentage of GDP expenditure and GDP, US$ per capita in 15 countries showed a significant correlation between those indicators, with the exception of Latvia and Estonia. Ten countries showed a positive correlation between these indicators with a correlation range from 0.47 in Bosnia and Herzegovina to 0.91 in the Republic of Serbia, meaning that an increase of THP is followed by an increase of GDP, US$. Five countries showed negative correlation, with a correlation range from −0.67 in Belarus to −0.94 in North Macedonia. Finally, private households' out-of-pocket payments on health as percentage of THE and GDP US$ per capita showed significant correlations in 14 out of 17 countries. Eight countries showed a positive correlation with a correlation range from 0.50 in Slovenia to 0.88 in Montenegro, meaning that OOP payments on health increase, as the GDP US per capita increases along. Six countries showed a negative correlation with a correlation range from −0.59 in Greece to −0.90 in Albania.

**Table 2:** Correlation between selected indicators: THE as % of GDP, OOP as % of THE, and GDP, US$ per capita for all 17 countries.

**Countries**	**THE and OOP**		**THE and GDP**		**OOP and GDP**	
	***r***	***P***	***r***	***p***	***r***	***P***
Ukraine	−0.06	0.79	0.59	0.01	0.26	0.28
Albania	0.87	0.00	−0.81	0.00	−0.90	0.00
Bosnia and Herzegovina	−0.24	0.32	0.47	0.04	−0.86	0.00
North Macedonia	0.66	0.00	−0.94	0.00	−0.76	0.00
Belarus	−0.35	0.13	−0.67	0.00	0.84	0.00
Bulgaria	0.86	0.00	0.63	0.00	0.79	0.00
Republic of Serbia	0.74	0.00	0.91	0.00	0.86	0.00
Romania	−0.64	0.00	0.79	0.00	−0.38	0.10
Montenegro	−0.82	0.00	−0.82	0.00	0.88	0.00
Russian Federation	0.51	0.02	0.57	0.01	0.83	0.00
Turkey	−0.79	0.00	0.64	0.00	−0.79	0.00
Latvia	−0.07	0.77	0.20	0.39	−0.70	0.00
Lithuania	0.12	0.63	0.64	0.00	0.60	0.01
Croatia	−0.63	0.00	0.69	0.00	−0.36	0.12
Estonia	−0.42	0.06	0.32	0.17	0.58	0.01
Slovenia	0.08	0.75	0.69	0.00	0.50	0.02
Greece	−0.64	0.00	0.86	0.00	−0.59	0.01

*r, Pearson's correlation coefficient; p, p values; THE—Total health expenditure as percentage of GDP expenditure; OOP—private households' out-of-pocket payments on health as percentage of THE; GDP—gross domestic product (GDP), US$ per capita*.

## Discussion

In the period from 1880 to 1990, data on the OECD countries showed similar rising paths in investing of GDP on health, with France being in the leading spot (22). Looking at the OECD countries nowadays, which are high-income countries, it can be noticed that they spend a large percentage of GDP on health. The United States is the country that spends the highest percentage of all countries in the OECD group, with almost 17% in 2018 (23). Comparing Balkan and East European Countries with the OECD group, it can be concluded that, first of all, they do not belong to the same income group, so one can expect that THE as % of GDP is much lower for these countries. However, Serbia is the country with the highest investment of all of these countries, with 10.37% in 2014. On the other hand, a larger share of GDP does not necessarily indicate the existence of a better health care system. The United States spends more on health care due to higher prices of health services, and not because of the greater use of those services (24).

Unfortunately, more than 80% of people living in low- and middle-income countries have benefited from only 20% of global health spending (25). The World Health Organization has divided countries into six regions according to what part of GDP is invested in health. Among them, the European region invested the most in the monitoring period, from 2000 to 2017, with 7.8% in the last considered year (26). Countries that were analyzed in our research belong to the European region.

In 2014, the global average level of GDP percentage spent on health was 6.1%, while the average value of all countries considered in our research for the same year was 7.2%, higher than the global average (26). From all of the European countries, Switzerland had the highest investments (12.3%), while Turkey (4.2%), Romania (5.25%), and Latvia and Lithuania (both 6.3%) had the lowest ones in 2017 (27, 28). Romania (5.25%) and Turkey (5.35%) had the lowest median percentage of GDP investments in health within the 17-year follow-up period, which is in line with previous research presenting these two countries as the ones with slowest growth (29). Similar results were found in Eurostat analysis, where Romania had the lowest investment of GDP in health, roughly 5%.

Governments provide an average of 51% of a health spending of countries, while more than 35% of health spending per country comes from out-of-pocket expenses (30). On the average, in 2015, 32% of health expenditure was out-of-pocket (31). More than half of countries in our analysis showed increase of OOP spending, with the highest percentage in the Russian Federation in which the OOP increased 2.7 times in 2014 in comparison to 1995. On the contrary, in Bosnia and Herzegovina, there was a decrease of 2.3 times in the analyzed period. Out-of-pocket share of health expenditure in 2016 was the highest in Switzerland, accounting for 30% (32).

Our results showed that more than half of the analyzed countries have OOP percent share higher than 30%, with Albania on the top with 50%, followed by Ukraine and Russia accounting for 46%. They are in line with the database “*Our world in data*” with these three countries as the ones with the highest OOP share, along with Bulgaria (33). Slovenia (11.8%) and Croatia (13.5%) showed the smallest percentage, similar to the United States.

The GDP per capita in the United States was recorded at the amount of US$53,356 in 2017 (34). Compared to any country in our analysis, the United States has far higher health spending that is almost twice as high as Slovenia's costs (US$23,594), and these costs are higher than in all other countries in our study.

Slovenia, in 2018, according to data from World Bank, had spending equal to US$26,234, ranking 36th. Estonia (US$22,928) and Greece (US$20,324) were listed as 41st and 42nd, respectively, while Croatia was 57th (US$14,869) (35). Looking at the data from our study, countries with the highest spending were Slovenia (US$23,594) on top, followed by Estonia (US$19,705) and Greece (US$18,613). Countries that had the least health spending were Ukraine (US$2640) and Albania (US$4538)—nine and five times smaller than Slovenia, respectively.

Out-of-pocket expenditures are also positively correlated with the GDP share spent on health. The OOP budget share is higher in countries that spend a large share of their GDP on health and lower in countries that channel more of their total health spending through social health insurance (36). Those results are in line with our findings where higher spending on GDP led to higher share of OOP spending in nine observed countries.

THEs rise along with the development of the country. Such development allows access to new pharmaceuticals, medical technology, and also a new point of view, in general. However, many medical decisions must be carefully considered because the inappropriate application of new technologies can lead to severe consequences for government funds and unnecessary costs for the citizens themselves, leading more to problems than to some desired solutions (37, 38). Rancic et al. showed that THE, expressed as percentage of GDP in the most of the selected Balkan countries, for the period from 1995 to 2012, had an obvious increase (39). THE share of GDP among observed countries in 2012 was highest in Albania, while Romania, Serbia, and Bulgaria recorded about 1.5-fold higher share in comparison to 1995. Our study shows that Bulgaria, Serbia, and Slovenia had the biggest progress.

During the last two decades, significant change in US dollar health expenditure per capita was observed, where, in 2000, average world spending on health was $472, and in 2016, it increased more than double, with $1026 (40, 41). Our analysis pointed out that Balkan and Southeastern countries have also shown an increased trend concerning per capita spending, with Slovenia and Greece at the top.

It is well known that OOP payment prevents some people from accessing needed care, while others face financial hardship when they access services. In 2000, percentage of OOP spending on health in the world was around 19, and in 2014, it decreased to about 18% (42). Our study considering Balkan and Southeastern countries showed that Albania, Ukraine, and the Russian Federation had greater difference from the world's average (average of percentage of OOP spending on health worldwide in 2014 was 18%).

The problem of population aging is one of the worrying problems in all countries where medical costs are rising, and such is the situation in the Balkans and Eastern Europe (43). Older people have more needs related to medicine and health systems that do not include these age groups adequately and will adversely affect both national and private budgets that need to cover that (44, 45). This problem is realistic, since older people suffer from more than one non-communicable disease or should be subjected to more than one surgical intervention accompanied by necessary laboratory analyses (46, 47). Also, the home care should not be forgotten, since this kind of health care is usually provided by family members (out-of-pocket expenditures) (48, 49).

Health systems are influenced by epidemiological transition and health financing, which affect the type of services needed. Health care expenditures are rising fast, faster than the global economy, becoming a huge global concern especially in the low- and middle-income countries (50, 51). WHO report on Universal Health Coverage issued on 2016 indicates that the world has spent almost 10% of GDP on health with an average per capita expenditure of US$1000.

More than 70% of health care costs relate to outpatient and inpatient treatment, medications, and medical supplies, which limits other types of care, such as prevention services or health administration services (52, 53). Recent findings about global burden of diseases and risk factors for them can be of large help in decision making concerning health expenditure rise and health investments (54, 55). These facts should be available for as much country as it is possible, especially concerning their economic level of development.

Health economy becomes of strong importance in the public health area, or even in the field of national policy. The results of studies in the field of economics, thanks to what they provide, can influence important decisions, such as the level of investment in health care, as part of GDP, and can help to solve existing problems in a better way (56, 57). Comparing the results with countries that have a similar health care system can help prevent the emergence of similar problems that are observed in those countries (58, 59). Increasing number of health care policymakers and managers has enabled health economics to become a tool for making allocation of resources more rational (60, 61).

## Conclusions

Our study compared macroeconomic and health expenditure indicators of interest between selected Balkan and Eastern European countries in the period from 1995 to 2014. The results show that Greece and Slovenia have the largest median values of GDP per capita throughout the whole period and the largest increment trend was in Lithuania. The median value in OOP payment of THE was the highest in Albania and Ukraine, while the largest decrease in trend was noticed in Albania and Bosnia and Herzegovina. Bosnia and Herzegovina and Greece had the largest median value of THE as percentage of GDP in the observed period, while regression trend analysis showed that Serbia had the largest increase. Most of the countries showed a significant correlation between the observed indicators.

## Data Availability Statement

The raw data supporting the conclusions of this article will be made available by the authors, without undue reservation.

## Author Contributions

MS, NR, MJ, and BV: conception of work. MS, MJ, and BV: design of the work. VD-S, NR, MS, BV, and SV: the acquisition and analysis. MS, NR, BV, VD-S, and SV: interpretation of data. NR, BV, MS, and VD-S: drafted the work and substantively revised it. All authors read and approved the final manuscript.

## Conflict of Interest

The authors declare that the research was conducted in the absence of any commercial or financial relationships that could be construed as a potential conflict of interest.

## Abbreviations

GDP: gross domestic product
OOP: out-of-pocket spending
THE: total health expenditure
HFA: Health for All.
